# Supplementary material for: The role of water mobility on water-responsive actuation of silk
Source: Nat Commun. 2024 Sep 27;15:8287. doi: 10.1038/s41467-024-52715-6 (PMC11436739; doi:10.1038/s41467-024-52715-6)
Supplement: Supplementary file 1 — Supplementary Information [file 41467_2024_52715_MOESM1_ESM.pdf]

# Supplementary Information for

**The role of water mobility on water-responsive actuation of silk**

**Authors:** Darjan Podbevšek[1,2], Yeojin Jung[1,2], Maheen K. Khan[1,2], Honghui Yu[3], Raymond S. Tu[1,2]*, and Xi Chen[1,2,4]*

**Affiliations:**

[1]Advanced Science Research Center (ASRC) at the Graduate Center, The City University of New York, 85 St. Nicholas Terrace, New York, NY, 10031 USA.

[2]Department of Chemical Engineering, The City College of New York, 275 Convent Ave., New York, NY, 10031 USA.

[3]Department of Mechanical Engineering, The City College of New York, 275 Convent Ave., New York, NY, 10031 USA.

[4]PhD Programs in Chemistry and Physics, The Graduate Center of the City University of New York, 365 5th Ave., New York, NY, 10016 USA.

*Correspondence to: xchen@gc.cuny.edu, tu@ccny.cuny.edu

**This PDF file includes:**

Supplementary Notes (1-2)
Supplementary Figures S1 to S13
Supplementary References (1-30)

**Supplementary Notes**

## 1. Contextualization of H-bonding water networks in materials

Nanoconfined water can be grouped into bound and mobile water. Bound water refers to water within short distances and with strong interactions with the material's surface, while mobile water includes both bulk-like and unbound hydration water[1–4]. The O-H stretch peak deconvolution and analysis was performed in various IR and Raman spectroscopy studies, as they both probe the same O-H stretch modes[5–18]. The ATR-FTIR study of gradual layering of water molecules on silica as a function of relative humidity (RH), presented distinct peak evolution, with the first layer showing ordered tetragonal orientation at 3230/cm and the subsequent outer layers presenting increasingly bulk-like behavior with the peak at 3400/cm[19]. Moreover, hydrophobic and hydrophilic functionalization of a silicon surface has shown different ratios between the structured (3200-3275/cm) and bulk water (3400-3450/cm)[20], with the hydrophilic surface forming up to two layers of bound water during hydration, while the hydrophobic surface forms clusters of only a single layer of structured water at the surface. A 3280/cm peak, similar to our identified peak at 3290/cm, corresponds to bound water in cellulose fiber interaction in nano-bridge crosslinks of cotton fibers[21]. Other FTIR studies show that modification to the H-bonding network yield measurable changes from bulk-like water for nano-confined systems, including water in minerals[22], mesoporous silica[23,24], carbon nanotubes[25] and porous coordination polymers[26].

## 2. Deconvolution of FTIR O-H stretching spectra

To determine the H-bond populations, we first performed the second derivative of the FTIR O-H stretching spectra obtained at both 10% RH and 90% RH (see Fig. S8a), and then compared these

second derivatives with the peaks reported previously in literature (Fig. S8b) [6,7,15,17,18,25,26,28–30]. It is clear that all our silk samples have three major H-bond populations at FTIR peaks of 3210, 3290 and 3410 /cm, which align well with H-bonds found in other materials (Sun, (2013)[7]) (Fig. S8b). Since our RSF samples still contain significant amounts of tightly bound and bulk-like mobile water, evident at both ends of the FTIR spectra, we then assigned two more peaks to represent these less well-defined edge populations. To represent tightly bound water, the 3000/cm peak was chosen as the second derivative characteristic of most RSF samples, representing the low wavenumber population. For bulk-like mobile water, the 3550/cm peak was identified by averaging the high-wavenumber peaks found with the second-order derivative analysis of the three samples at 10% and 90% RH (Fig. S8b). The 3550/cm peak is also fairly close to the previously reported peaks of 3540/cm (Ichii *et al.*[26], Mallamace *et al.*[15] and Laurson *et al.*[18]) and 3569/cm (Bernardina *et al.*[25]) (Fig. S8b).

In addition, we have also deconvoluted the O-H stretching band using both three and six populations, as follows.

Three-population deconvolution:

While using the three water populations (3295, 3460 and 3590/cm) suggested in the literature (Brubach *et al.*[17]), we observed that the deconvolution did not adequately fit the FTIR data (average $R^2$ 0.98768 +/-0.01013 for Silk-H, 0.98234 +/-0.00969 for Silk-M and 0.942885 +/-0.00897 for Silk-L), particularly at lower wavenumbers (Fig. S9). Whereas the five-population deconvolution yields a much better fit, with average $R^2$ values of 0.99928 for Silk-H, 0.99935 for Silk-M and 0.99735 for Silk-L. It is most likely because our RSF samples contain a significant amount of bound water, which increases the spectral intensity in the ~2800-3100/cm region. The

three-population deconvolution seems to be adequate only for Silk-H at 90% RH, but not for other RSF samples with higher levels of bound water.

Six-population deconvolution:

We have also deconvoluted the O-H stretching band using six-populations, with the addition free-O-H stretching peak of 3636/cm found in bulk water (Fig. S10 & Fig. S11). The addition of the 3636/cm peak does not contribute to the analysis, except for a 0.85% contribution to the total OH-peak area in the case of Silk-L at 10% RH (Fig. S10b). The negligible contribution of the free-OH stretching band to the deconvolution suggests that RSF samples contain a minimal amount of free-OH bonds.

In summary, the five-population deconvolution is the most reasonable approximation for our RSF samples, based on a combination of second derivative analysis of the FTIR spectra and the overlap of the peak positions found in literature. The three-population deconvolution underperformed in terms of fit quality, while the six-population deconvolution performed similarly to the five-population deconvolution, but the additional free-OH stretching peak did not yield any measurable contribution.

## Supplementary Figures

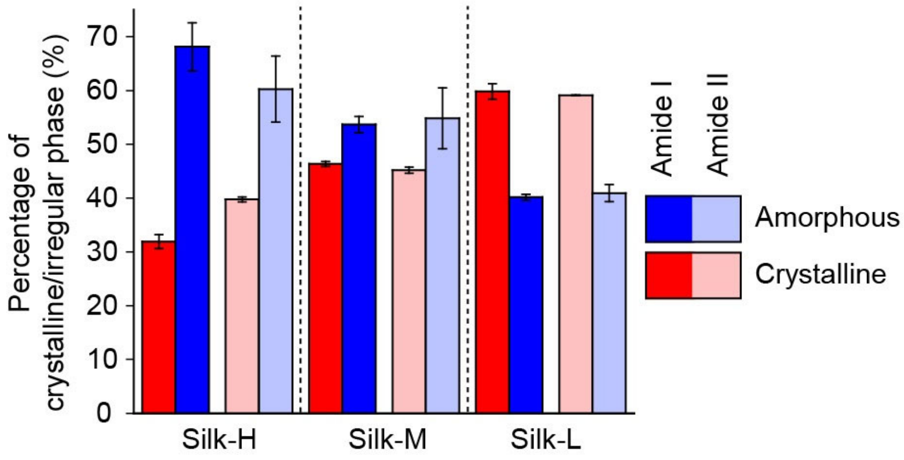


**Fig. S1 | Crystalline/Amorphous secondary structure from Amide I and II peak deconvolution.** Comparison of secondary structure (crystalline and amorphous phase) from the deconvolution of amide I and II peaks from the three regenerated silk fibroin samples with different post-treatment. In accordance with the established protocol[27], only Amide I peak is used to determine the secondary structure composition of the samples. Data are presented as the deconvolution peak area ± SE of the fit.

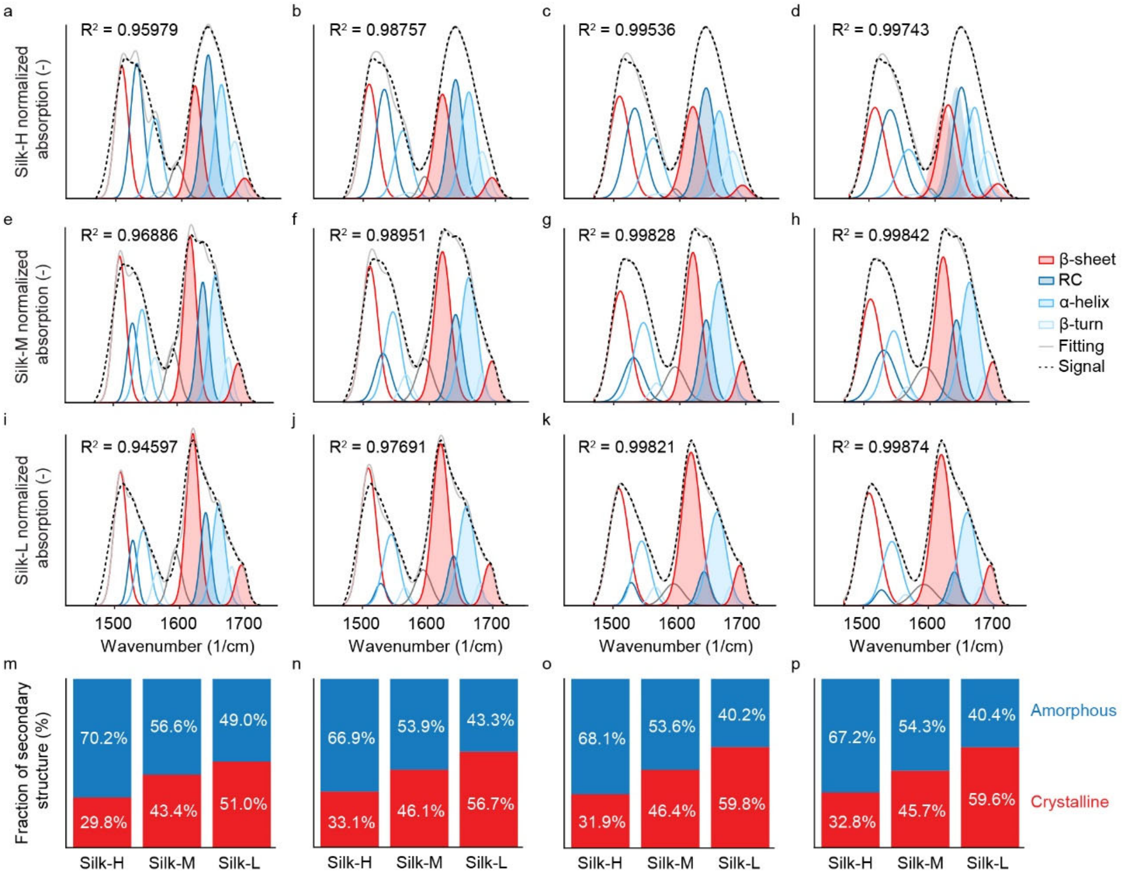


**Fig. S2 | Estimated amorphous and crystalline fractions with changing FWHM.** The lower limit of FWHM is fixed to 10/cm, and the upper limits vary for each column: **a**, **e**, **i**, **m**) 20/cm, **b**, **f**, **j**, **n**) 25/cm, **c**, **g**, **k**, **o**) 30/cm (value used for analysis) and **d**, **h**, **l**, **p**) 35/cm. The lower row of graphs, **m**, **n**, **o**, **p**) show proportional areas of the crystalline (β-sheet – red shaded peaks) and amorphous (RC, α-helix and β-turn – blue shaded peaks) contents of Amide I peak deconvolution.

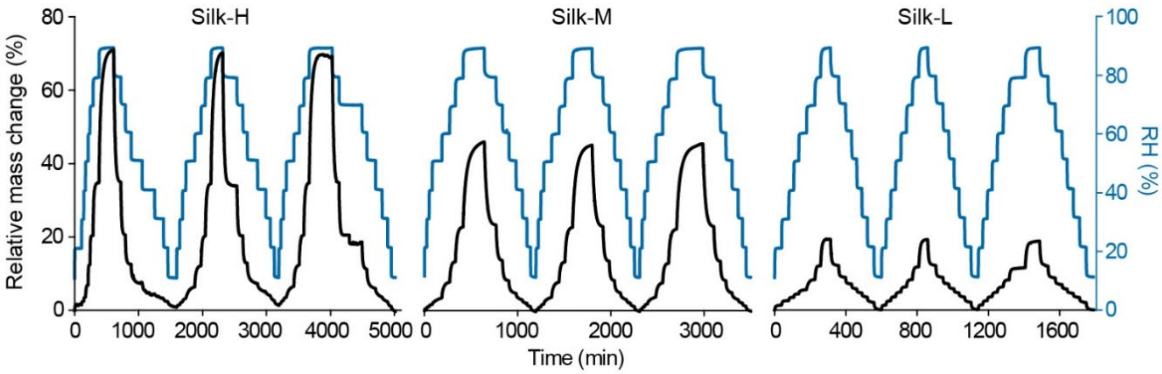


**Fig. S3 | Relative mass change and RH as a function of time measured by DVS for all three RSF samples.**

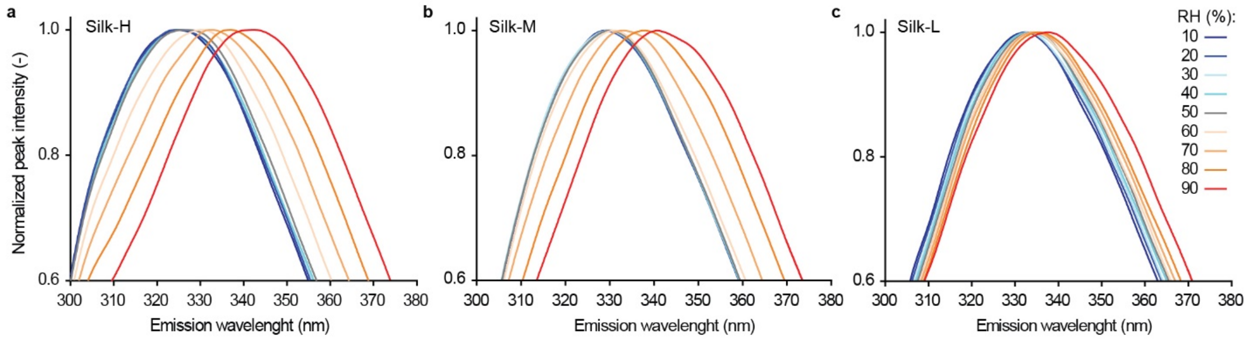


**Fig. S4 | Tryptophan fluorescence emission peak at 290nm excitation. a**) Silk-H, **b**) Silk-M and **c**) Silk-L emission shift of normalized inherent fluorescence from regenerated silk fibroin during hydration from 10% to 90% RH.

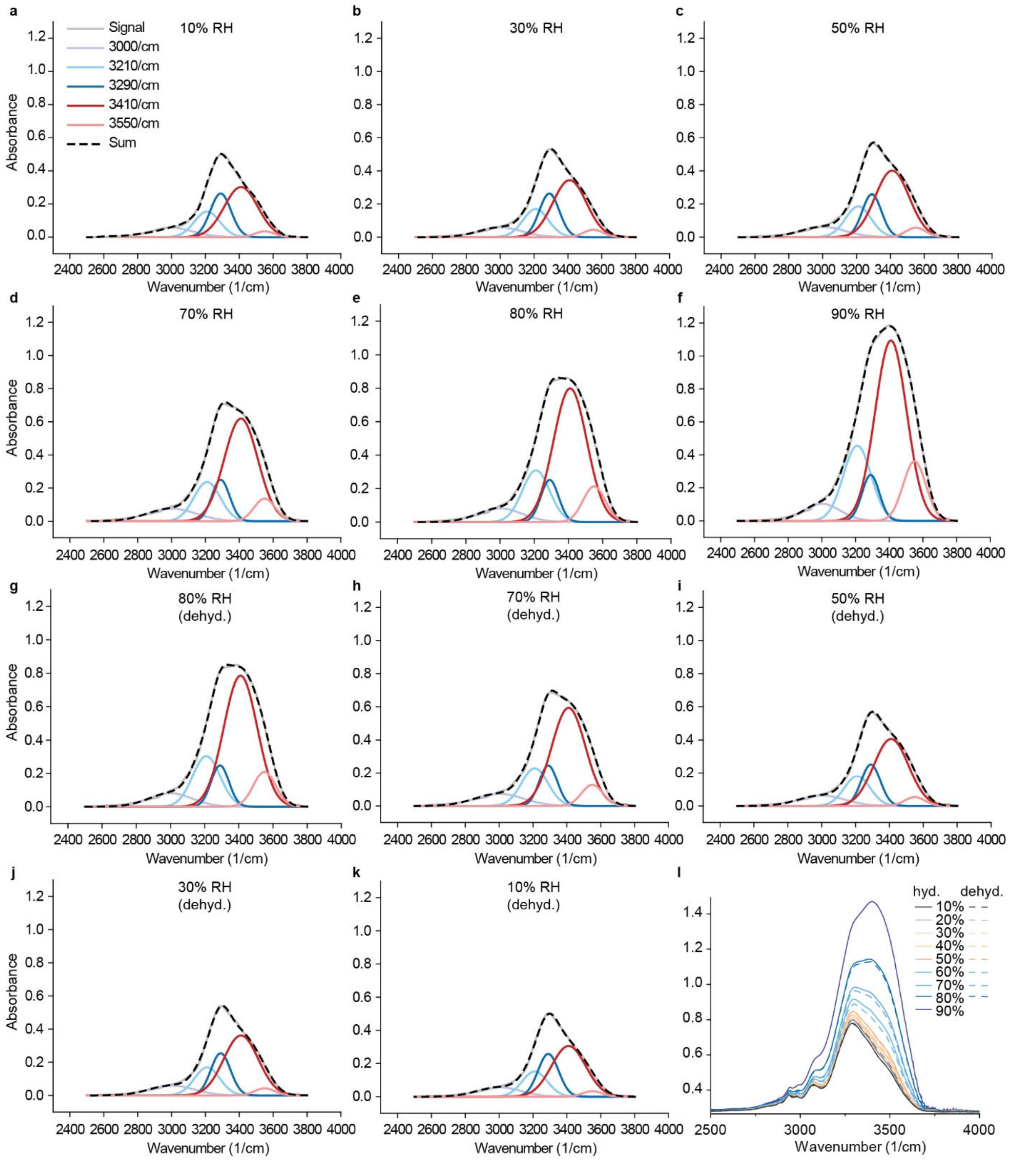


**Fig. S5 | The FTIR spectra of the Silk-H sample through hydration/dehydration cycle RH.** **a-k**) Individual treated absorption curves and the 5-peak deconvolution at 10%, 30%, 50%, 70%, 80%, and 90% RH (hydration & dehydration). **l**) The raw absorption curves from the humidity-controlled absorption FTIR experiment.

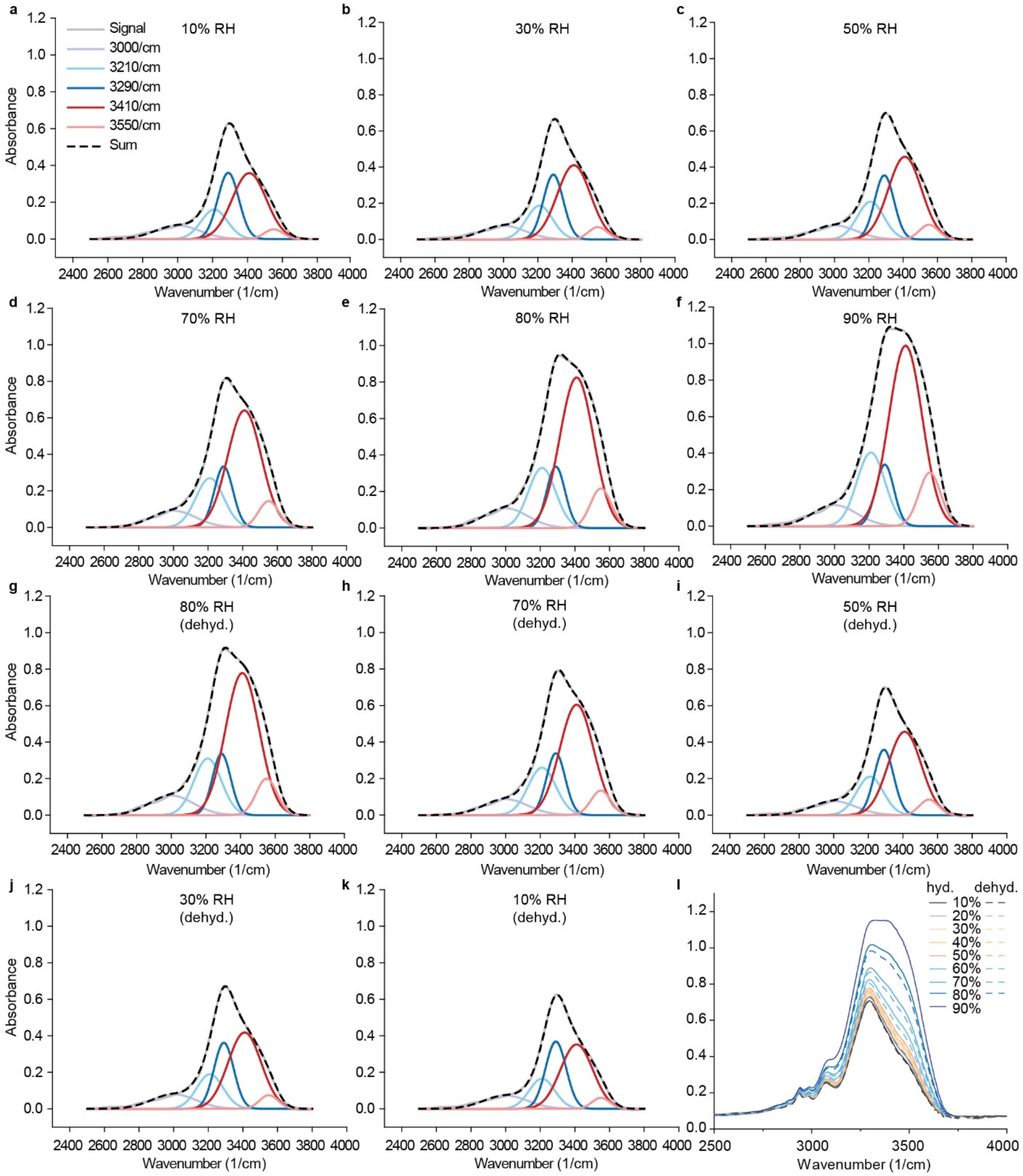


**Fig. S6 | The FTIR spectra of the Silk-M sample through hydration/dehydration cycle RH. a-k**) Individual treated absorption curves and the 5-peak deconvolution at 10%, 30%, 50%, 70%, 80%, and 90% RH (hydration & dehydration). **l**) The raw absorption curves from the humidity-controlled absorption FTIR experiment.

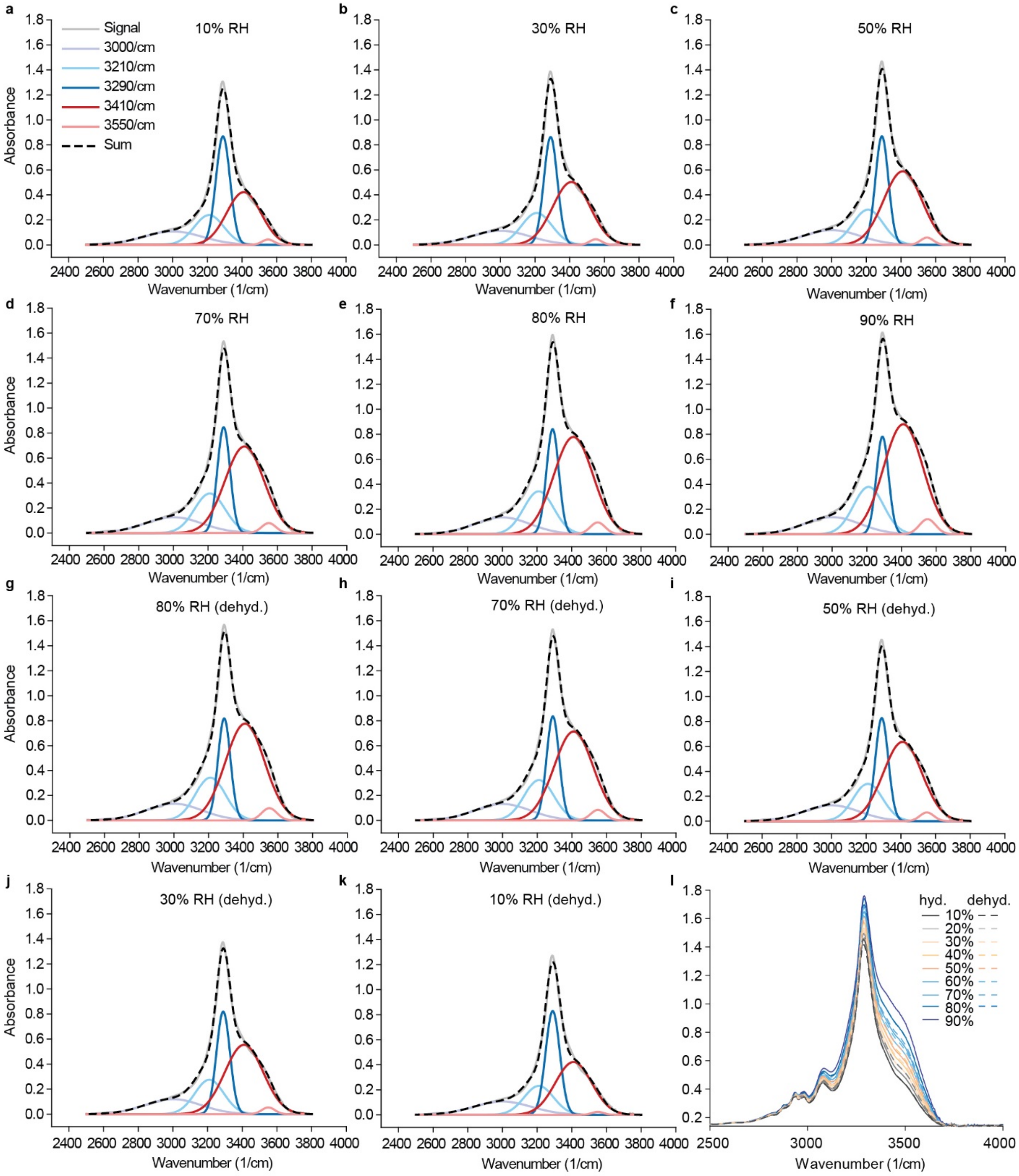


**Fig. S7 | The FTIR spectra of the Silk-L sample through hydration/dehydration cycle RH. a-k**) Individual treated absorption curves and the 5-peak deconvolution at 10%, 30%, 50%, 70%, 80%, and 90% RH (hydration & dehydration). **l**) The raw absorption curves from the humidity-controlled absorption FTIR experiment.

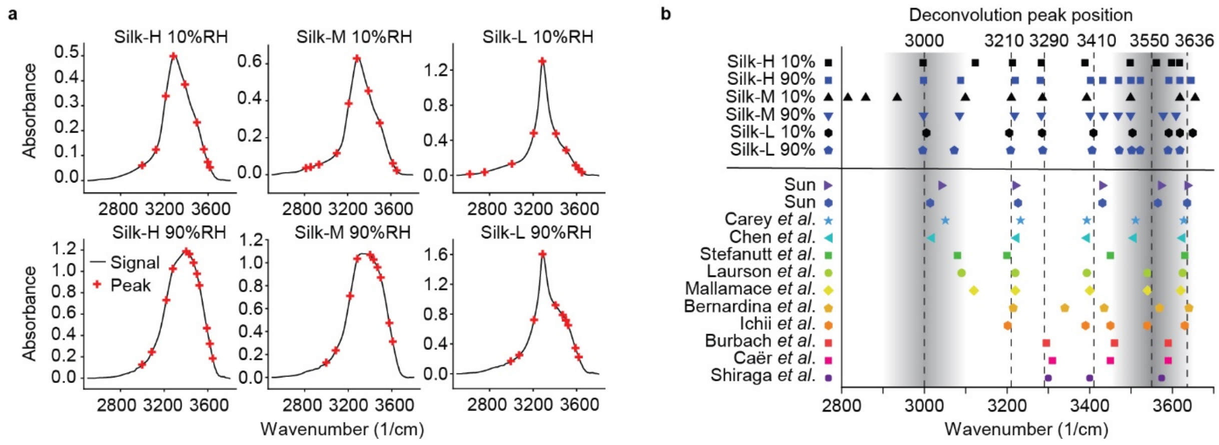


**Fig. S8 | Deconvolution peak position determination. a**) Peak wavenumber and intensity positions of 10 and 90% RH for all three RSF samples obtained through second-order derivative analysis of the OH-stretch peak. **b**) Comparison of second-order derivative analysis of the sample peaks with the peak positions found in literature[6,7,15,17,18,25,26,28–30] for bulk and nanoconfined water. The peaks chosen for the deconvolution are 3000, 3210, 3290, 3410 and 3550/cm. The 3000 and the 3550/cm peaks encompass several populations on the edge of the spectra.

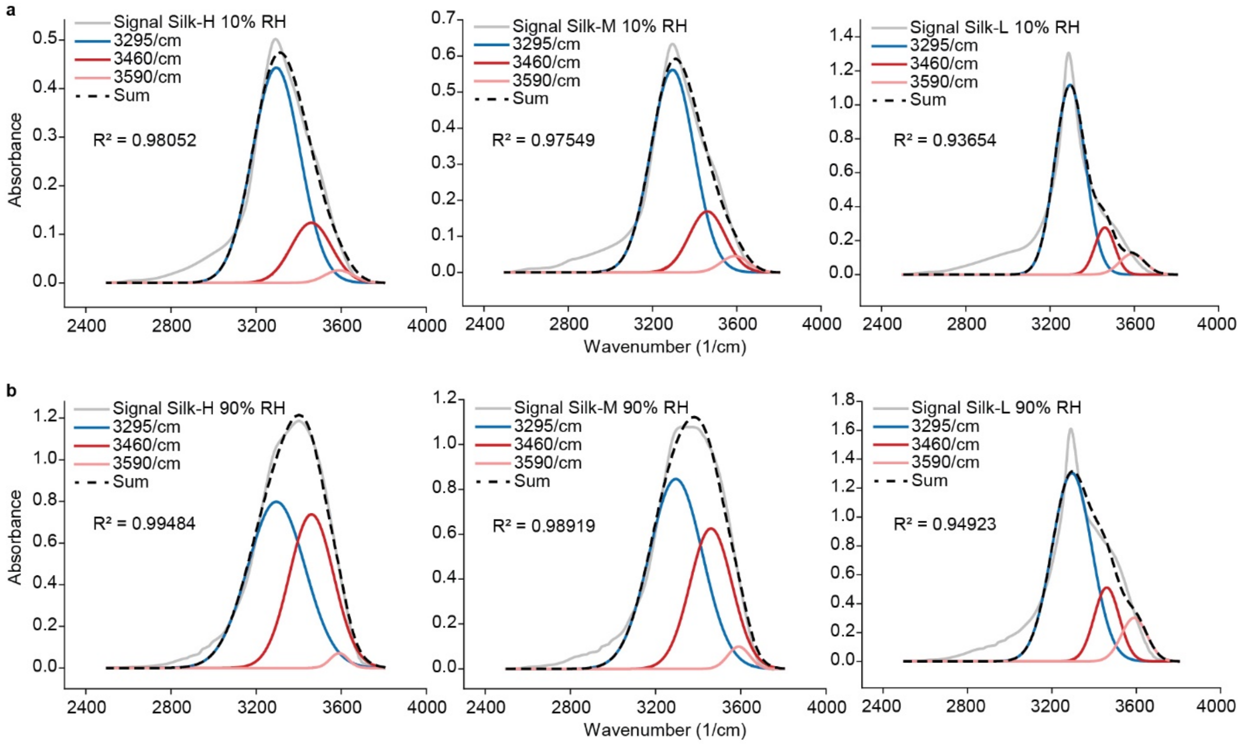


**Fig. S9 | The deconvolution of FTIR spectra of the Silk-H, Silk-M & Silk-L samples with peak positions from Brubach *et al*.[17] by 3-peak deconvolution. a**) at 10% RH. **b**) at 90% RH.

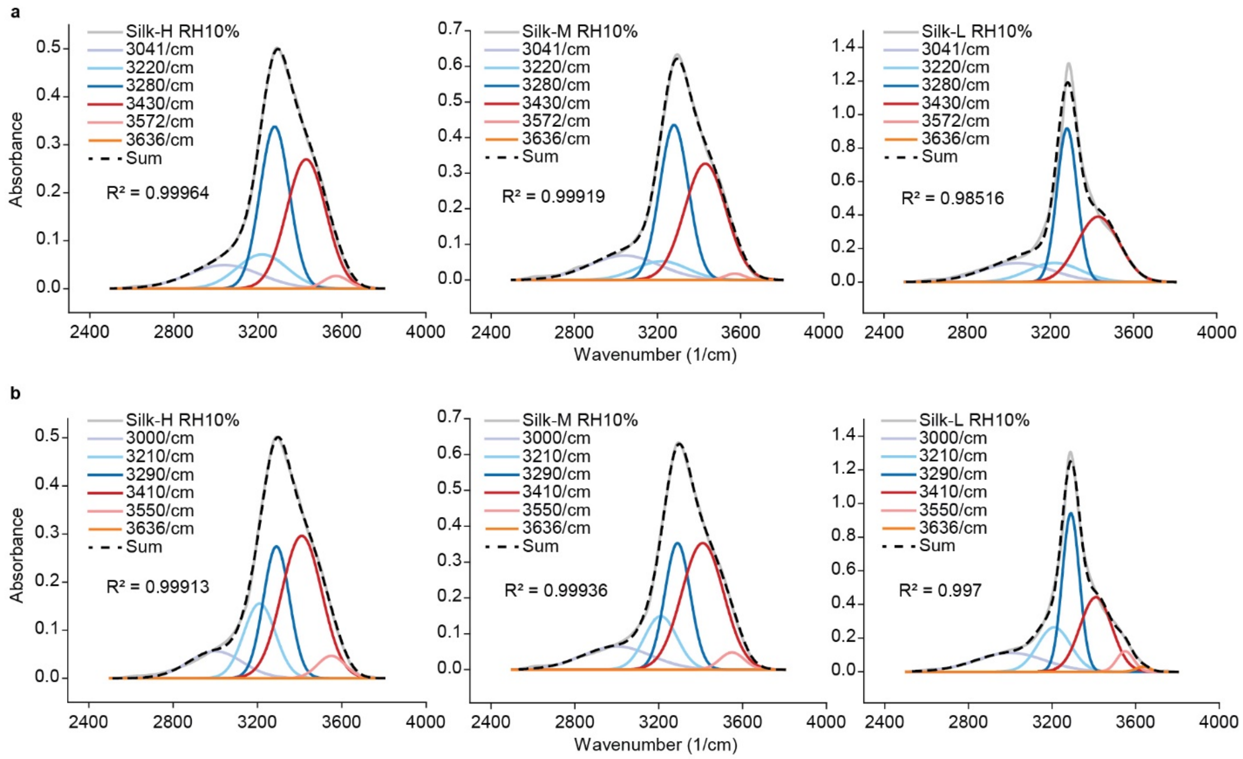


**Fig. S10 | Deconvolution of all three RSF samples at 10% RH with the two peak position protocols. a**) 6-peak deconvolution with peak position as found in Sun (2013)[7] and Igarashi et al. (2020)[21]. **b**) 6-peak deconvolution with peak position from our 5-peak deconvolution with the additional 3636/cm peak.

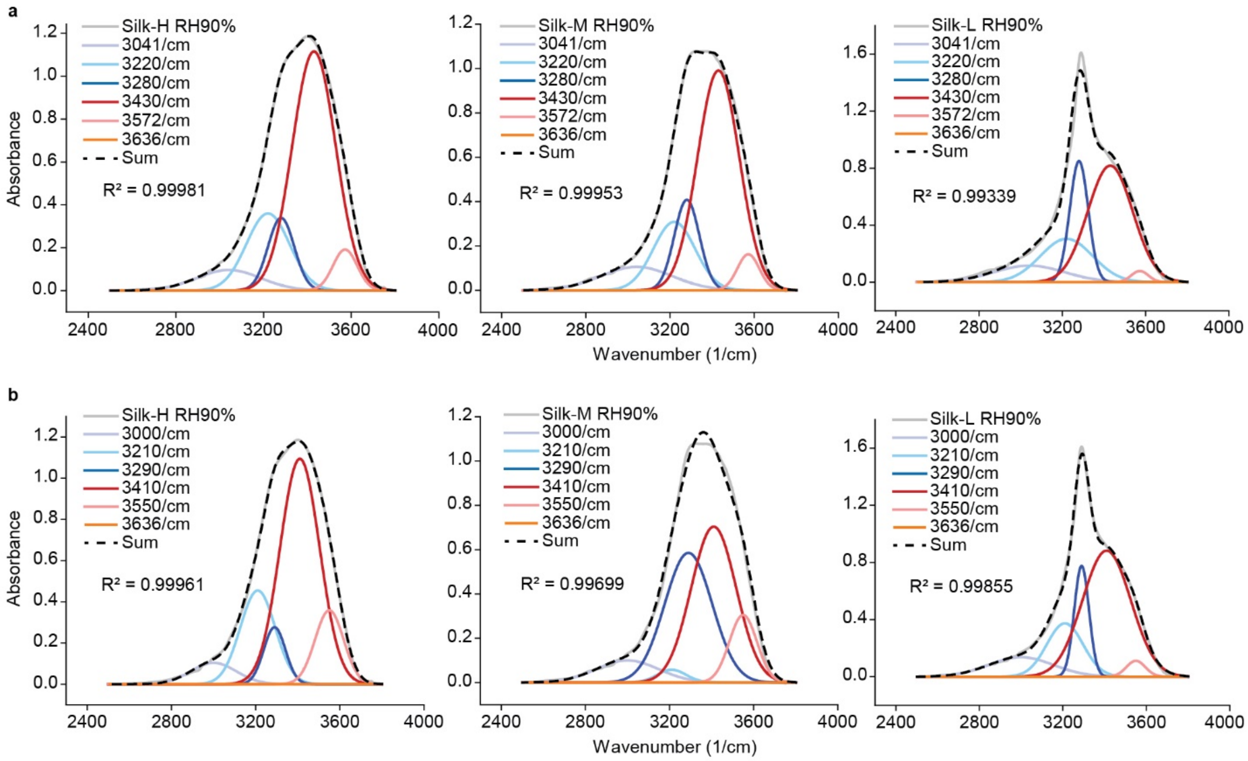


**Fig. S11 | Deconvolution of all three samples at 90% RH with the two peak position protocols. a**) 6-peak deconvolution with peak position as found in Sun (2013)[7] and Igarashi et al. (2020)[21]. **b**) 6-peak deconvolution with peak position from our 5-peak deconvolution with the additional 3636/cm peak.

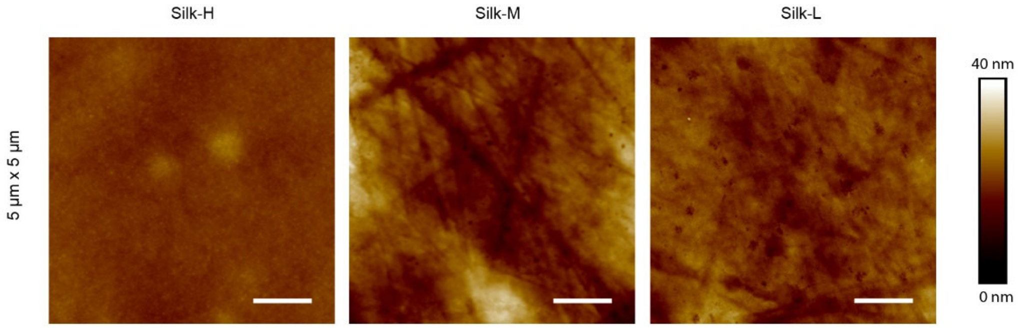


**Fig. S12 | AFM topographies of the RSF samples.** Scale bar is 1µm.

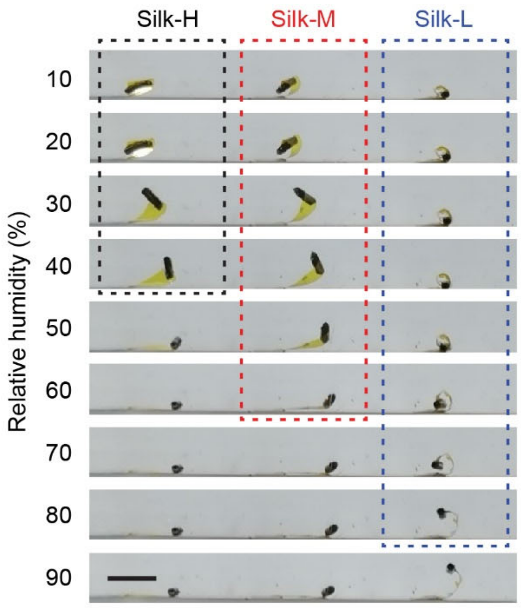


**Fig. S13 | WR actuation of RSF/polyimide bilayers.** RSF/polyimide bilayers started to actuate at different RH levels during dehydration. The scale bar is 5 mm.

## Supplementary References


1. Asakura, T. *et al.* Characterization of water in hydrated Bombyx mori silk fibroin fiber and films by 2H NMR relaxation and 13C solid state NMR. *Acta Biomater.* **50**, 322–333 (2017).
2. Hu, X., Kaplan, D. & Cebe, P. Effect of water on the thermal properties of silk fibroin. *Thermochim. Acta* **461**, 137–144 (2007).
3. Chen, S.-H. *et al.* Observation of fragile-to-strong dynamic crossover in protein hydration water. *Proc. Natl. Acad. Sci.* **103**, 9012–9016 (2006).
4. Calero, C. & Franzese, G. Membranes with different hydration levels: The interface between *bound* and *unbound* hydration water. *J. Mol. Liq.* **273**, 488–496 (2019).
5. Sun, Q. The Raman OH stretching bands of liquid water. *Vib. Spectrosc.* **51**, 213–217 (2009).
6. Sun, Q. The single donator-single acceptor hydrogen bonding structure in water probed by Raman spectroscopy. *J. Chem. Phys.* **132**, 054507 (2010).
7. Sun, Q. Local statistical interpretation for water structure. *Chem. Phys. Lett.* **568–569**, 90–94 (2013).
8. Hu, Q., Zhao, H. & Ouyang, S. Understanding water structure from Raman spectra of isotopic substitution H2O/D2O up to 573 K. *Phys. Chem. Chem. Phys.* **19**, 21540–21547 (2017).
9. Auer, B. M. & Skinner, J. L. IR and Raman spectra of liquid water: Theory and interpretation. *J. Chem. Phys.* **128**, 224511 (2008).

10. Nishiyama, H. *et al.* Chemical States of Water Molecules Distributed Inside a Proton Exchange Membrane of a Running Fuel Cell Studied by Operando Coherent Anti-Stokes Raman Scattering Spectroscopy. *J. Phys. Chem. C* **124**, 9703–9711 (2020).
11. Hofmann, D. W. M. *et al.* Investigation of Water Structure in Nafion Membranes by Infrared Spectroscopy and Molecular Dynamics Simulation. *J. Phys. Chem. B* **113**, 632–639 (2009).
12. Ferrari, M.-C., Catalano, J., Giacinti Baschetti, M., De Angelis, M. G. & Sarti, G. C. FTIR-ATR Study of Water Distribution in a Short-Side-Chain PFSI Membrane. *Macromolecules* **45**, 1901–1912 (2012).
13. Awatani, T., Midorikawa, H., Kojima, N., Ye, J. & Marcott, C. Morphology of water transport channels and hydrophobic clusters in Nafion from high spatial resolution AFM-IR spectroscopy and imaging. *Electrochem. Commun.* **30**, 5–8 (2013).
14. Arsov, Z., Rappolt, M. & Grdadolnik, J. Weakened Hydrogen Bonds in Water Confined between Lipid Bilayers: The Existence of a Long-Range Attractive Hydration Force. *ChemPhysChem* **10**, 1438–1441 (2009).
15. Mallamace, F. *et al.* Evidence of the existence of the low-density liquid phase in supercooled, confined water. *Proc. Natl. Acad. Sci.* **104**, 424–428 (2007).
16. Wolf, S., Freier, E., Cui, Q. & Gerwert, K. Infrared spectral marker bands characterizing a transient water wire inside a hydrophobic membrane protein. *J. Chem. Phys.* **141**, 22D524 (2014).
17. Brubach, J.-B., Mermet, A., Filabozzi, A., Gerschel, A. & Roy, P. Signatures of the hydrogen bonding in the infrared bands of water. *J. Chem. Phys.* **122**, 184509 (2005).

18. Laurson, P., Raudsepp, P., Kaldmäe, H., Kikas, A. & Mäeorg, U. The deconvolution of FTIR-ATR spectra to five Gaussians for detection of small changes in plant–water clusters. *AIP Adv.* **10**, 085214 (2020).

19. Asay, D. B. & Kim, S. H. Evolution of the Adsorbed Water Layer Structure on Silicon Oxide at Room Temperature. *J. Phys. Chem. B* **109**, 16760–16763 (2005).

20. Chen, L., He, X., Liu, H., Qian, L. & Kim, S. H. Water Adsorption on Hydrophilic and Hydrophobic Surfaces of Silicon. *J. Phys. Chem. C* **122**, 11385–11391 (2018).

21. Igarashi, T., Hoshi, M., Nakamura, K., Kaharu, T. & Murata, K. Direct Observation of Bound Water on Cotton Surfaces by Atomic Force Microscopy and Atomic Force Microscopy–Infrared Spectroscopy. *J. Phys. Chem. C* **124**, 4196–4201 (2020).

22. Yeşilbaş, M. & Boily, J.-F. Particle Size Controls on Water Adsorption and Condensation Regimes at Mineral Surfaces. *Sci. Rep.* **6**, 32136 (2016).

23. Knight, A. W., Kalugin, N. G., Coker, E. & Ilgen, A. G. Water properties under nano-scale confinement. *Sci. Rep.* **9**, 8246 (2019).

24. Baumgartner, B. *et al.* Pore Size-Dependent Structure of Confined Water in Mesoporous Silica Films from Water Adsorption/Desorption Using ATR–FTIR Spectroscopy. *Langmuir* **35**, 11986–11994 (2019).

25. Dalla Bernardina, S. *et al.* Water in Carbon Nanotubes: The Peculiar Hydrogen Bond Network Revealed by Infrared Spectroscopy. *J. Am. Chem. Soc.* **138**, 10437–10443 (2016).

26. Ichii, T. *et al.* Observation of an exotic state of water in the hydrophilic nanospace of porous coordination polymers. *Commun. Chem.* **3**, 1–6 (2020).

27. Belton, D. J., Plowright, R., Kaplan, D. L. & Perry, C. C. A robust spectroscopic method for the determination of protein conformational composition – Application to the annealing of silk. *Acta Biomater.* **73**, 355–364 (2018).
28. Chen, H.-C. *et al.* Innovative strategy with potential to increase hemodialysis efficiency and safety. *Sci. Rep.* **4**, 4425 (2014).
29. Caër, S. L. *et al.* A trapped water network in nanoporous material: the role of interfaces. *Phys. Chem. Chem. Phys.* **13**, 17658–17666 (2011).
30. Shiraga, K., Ogawa, Y. & Kondo, N. Hydrogen Bond Network of Water around Protein Investigated with Terahertz and Infrared Spectroscopy. *Biophys. J.* **111**, 2629–2641 (2016).
